# Acid-catalysed α-O-4 aryl-ether bond cleavage in methanol/(aqueous) ethanol: understanding depolymerisation of a lignin model compound during organosolv pretreatment

**DOI:** 10.1038/s41598-020-67787-9

**Published:** 2020-07-06

**Authors:** Edita Jasiukaitytė-Grojzdek, Matej Huš, Miha Grilc, Blaž Likozar

**Affiliations:** Department of Catalysis and Chemical Reaction Engineering, National Institute of Chemistry, Hajdrihova 19, 1000 Ljubljana, Slovenia

**Keywords:** Sustainability, Bioalcohols

## Abstract

The selective lignin conversion into bio-based organic mono-aromatics is a major general challenge due to complex structure itself/additional macromolecule modifications, caused by the cleavage of the ether chemical bonds during the lignocellulosic biomass organosolv pulping in acidified aqueous ethanol. Herein, the acido-lysis of connected benzyl phenyl (BPE), being a representative model compound with α-O-4 linkage, was investigated in methanol, EtOH and 75 vol% EtOH/water mixture solutions, progressing each time with protonating sulfuric acid. The effect of the physical solvent properties, acidity of the reaction process media and temperature on rate was determined. Experiments suggested BPE following S_N_1 mechanism due to the formation of a stable primary carbocation/polarity. The product species distribution in non-aqueous functional alcohols was strongly affected. The addition of H_2_O was advantageous, especially for alkoxylation. Yield was reduced by a factor of 3, consequently preserving reactive hydroxyl group. Quantitative experimental results indicated key performance parameters to achieve optimum. Organosolv lignins were further isolated under significantly moderate conditions. Consecutive structural differences observed supported findings, obtained when using BPE. H_2_O presence was again found to grant a higher measured –OH content. Mechanistic pathway analysis thus represents the first step when continuing to kinetics, structure–activity relationships or bio-refining industrial resources.

## Introduction

Serious concerns about the use of fossils fuels require the development of new procedures for the production of alternative fuels from sustainable, renewable and non-food resources. Lignocellulosic (LC) biomass represents one of low-cost, abundant and renewable materials with a high potential for the conversion into numerous chemicals^1^ or precursors for polymer synthesis^2^. The major components of the LC biomass are cellulose, hemicellulose and lignin. Lignin is an amorphous three-dimensional polymer of phenylpropanoid units interconnected by ether (α-O-4; β-O-4) and carbon–carbon (C–C) bonds^3^. Due to its aromatic, highly functionalized structure and abundancy, lignin is considered to be a very promising renewable and sustainable feedstock for the production of the chemicals, especially bio-aromatics^4,5^.

The conversion of lignin into value-added chemicals involves three steps: isolation, depolymerisation and final upgrading of the obtained platform chemicals. The fractionation procedure is essential as it determines the structure and reactivity of the isolated lignin. The content of β-O-4 bonds in the lignin molecule is besides the low molecular weight and low dispersity one of the key parameters, defining the suitability of lignin for further conversion into the value-added chemicals by depolymerizing and upgrading uniform lignin fragments. In order to separate lignin, the LC biomass is usually treated with acidified organic solvent and/or water as a co-solvent^6,7^. During fractionation in acidified media, lignin undergoes several structural changes due to the cleavage of aryl-ether bonds, followed by the substitution and/or condensation leading to the formation the lignin structures less susceptible to the depolymerisation^8^. An efficient lignin depolymerisation has been a major challenge for the last decade, having led to the development of new LC biomass fractionation techniques, such as the ‘lignin-first’ approach^9^ or lignin isolation through the formaldehyde stabilization^10^.

For depolymerisation by direct hydrogenolysis in the LC biomass (‘lignin-first’ approach), a heterogeneous metal catalyst is usually mixed with solid biomass in a high-pressure batch reactor, skipping the isolation of lignin. Despite the high efficiency of the ‘lignin-first’ approach to isolate lignin and concurrently produce aromatic monomers, some issues such as catalyst recovery and mass transfer limitations have limited the up-scale of this methodology. An outstanding lignin isolation/depolymerisation strategy using the formaldehyde stabilization/hydrogenolysis has been reported by Luterbacher and co-workers^10^. The addition of formaldehyde prevented the condensation of lignin by forming 1,3-dioxane/acetal structures with α- and γ-hydroxyl groups in the lignin side-chain and by functionalizing aromatic rings to generate hydroxymethyl groups. A highly efficient depolymerisation of formaldehyde-treated lignin by hydrogenolysis has the potential to have a significant impact on the shift from the petroleum-based to sustainable, biomass-based society.

LC biomass fractionation using the organosolv process has been recognized as environmentally friendly, yielding ether bond-rich lignin of higher purity compared to the lignin obtained with other methods, which is essential for its valorisation into value-added chemicals^11^. However, the main advantage of the organosolv process is its efficient fractionation of separate streams of major biomass components (cellulose, lignin and hemicelluloses), thus allowing the valorisation of all components. Cellulose and lignin are recovered as solids, while hemicelluloses and sugar degradation products are recovered from the water-soluble fraction as furfural and hydroxymethylfurfural^12^. The most common organosolv pretreatment systems apply aqueous ethanol or methanol with mineral acids as catalysts. Additionally, a low cost, low boiling point, easy recovery and green production make ethanol and methanol very attractive solvents for the organosolv pretreatment. Ethanol is a suitable solvent as its eventual losses in the reactive solvolysis could be compensated by feeding the fermentation-based bioethanol where bio-based ethanol could be collected in the product^13^. Also considering the maturity of the bioethanol production process from LC biomass using the organosolv process, the optimization of the isolated lignin quality would facilitate lignin valorisation, thus improving the efficiency of a complete organosolv-process-based biorefinery. To optimize the quality of the isolated lignin in terms of the preserved ether bonds, it is essential to understand the mechanism of the ether bond cleavage and the effects of operating conditions.

The most abundant ether bonds in lignin are α-O-4 (6 – 8 %), β-O-4 (45 – 60 %), 4-O-5 (4 – 9 %), β-1 (7 – 9 %), and 5–5 (3 – 27 %) linkages^14,15^. During organosolv pretreatment, lignin-carbohydrate and α-O-4 bonds in the lignin macromolecule are broken predominantly, while the scission of β-O-4 linkages occurs to a relatively smaller extent^16^. A relatively lower activation energy for the hydrolysis of α-O-4 bond (80 – 118 kJ mol^–1^)^17^ than the one for β-O-4 bond hydrolysis (150 kJ mol^–1^)^18^ and accordingly a much faster hydrolysis of α-aryl ethers was demonstrated in several studies^19,20,21^. Furthermore, the predominance of the cleavage of α-O-4 bonds during ethanol organosolv hydrolysis of *Miscanthus* reported by Hagea et al*.*^20^ indicated that β-O-4 cleavage is not the controlling reaction and did not play a significant role in the delignification process, while in Alcell lignin β-O-4 structures were even found to survive chemical processing^22^.

In numerous studies benzyl phenyl ether (BPE) was used as a α-O-4 linkage model compound to explore mechanisms of catalytic α-ther bond cleavage in aqueous and apolar phases^23,24,25^, in supercritical water and supercritical methanol^26^ because it has a weak ether bond (bond dissociation energy 215 kJ mol^−1^) and belongs to the most labile ether bonds in lignin^27^. In addition, α-O-4 linkage represents a typical lignin-carbohydrate bond, which has to be cleaved to liberate lignin from the lignocellulosic matrix^28^. Accordingly, benzyl phenyl ether was used as a α-O-4 linkage model compound to study the cleavage of one of the common bonds in the lignin structure in this work. We investigated the reaction pathway of aryl-ether bond cleavage in various solvents, used for organosolv pretreatment, such as ethanol and methanol. Additionally, the effects of water when used as a co-solvent and operating conditions on to the reaction and the product distribution were also studied.

Acidolysis of benzyl phenyl ether, being an important α-O-4 linkage model compound, has been investigated in ethanol, methanol and in 75 vol% ethanol/water with sulphuric acid. Here, we seek to understand the effect of alcohols, commonly used for the organosolv process, on the extent of the ether bond cleavage and alkoxylation reactions in aqueous and non-aqueous reaction media. Furthermore, the impact of the reaction media acidity and temperature on the product distribution (reflecting the conversion and selectivity of ongoing reactions) over time was studied.

## Material and methods

### Materials

All the reactants, gases, solvents and external calibration standards were of reagent grade and were used without further purification, specifically: benzyl phenyl ether (98 wt%, Tokyo Chemical Industry co LTD, Tokyo, Japan, CAS number 946-80-5), phenol (99.5 wt%, Carlo Erba Reagents SAS, Val de Reuil Cedex, CAS number 108-95-2), benzyl alcohol (≥ 99.0 wt%, Fluka Chemie GmbH, Buchs, Swizerland, CAS number 100-51-6), 2-benzylphenol (≥ 98.0 wt%, Sigma Aldrich, St. Louis, MO, USA, CAS number 28994-41-4), 4-methylbenzyl alcohol (98 wt% Sigma Aldrich, St. Louis, MO, USA, CAS number 589-18-4), ethanol (EtOH, absolute anhydrous, Carlo Erba Reagents SAS, Val de Reuil Cedex, France, CAS number 64-17-5), methanol (MeOH, ≥ 99 %, for HPLC, CAS number 67-56-1), sulfuric acid (H_2_SO_4_, 95 – 97 % Merck, Darmstad, Germany, CAS number 7664-93-9), nitrogen (5.0, Messer, Bad Soden am Taunus, Germany).

### Acidolysis experiments

Acidolysis was carried out in a six-parallel batch high-pressure reactor system, consisting of vessels with 250 mL volume, equipped with a magnetically driven Rushton turbine impeller (Amar Equipment Pvt. Ltd, Mumbai, India). Each reaction vessel was filled with the reactant (benzyl phenyl ether: BPE), the solvent (ethanol, methanol) or the solvent mixture (ethanol/water) and sulfuric acid. The reactor was placed in a housing, sealed, flushed twice and pressurized with nitrogen up to 1 MPa. All trials were performed with the stirring speed of 600 min^–1^. The heating was set to start at room temperature, gradually increase and kept at the set temperature for 4 h. The reaction was quenched by rapidly cooling down the reactor. Before opening the autoclave, the gaseous phase was released and the headspace was purged with nitrogen. The operating conditions such as time, temperature and pressure were automatically recorded by a Scada system. The experiments performed are summarized in Supplementary Table S1 and can be found online.

Here, BPE was used as the reactant throughout. BPE was dissolved in a solvent (ethanol, methanol) or solvent mixture (75 vol% ethanol/water) in the ratio of 1 : 70 (w/v) making 120 mL of total reaction volume. Experiments were performed at various temperatures (160 – 200 °C) and with various catalyst loadings 0.5 – 1.5 % of H_2_SO_4_ based on dry matter (for 1 g of model compound 0.025 – 0.075 g of H_2_SO_4_ in form of 2 M solution were used, thus taking into account that wood contains approximately 20 % of lignin).

Samples were collected during the reaction in a following order: the first sample was taken at room temperature, the second one halfway up the heating ramp, the third one at the final reaction temperature and then every 30 min until the end of the treatment. Eleven samples of 1 mL were collected for each experiment.

### GC–MS analysis

The samples were analysed using gas chromatography coupled with mass spectrometry (GC–MS; 2010 Ultra, Shimadzu, Kyoto, Japan) with an additional FID (flame ionization detector) detector, equipped with the Zebron ZB-5 (Phenomenex, Torrance, CA, USA) 60 m × 0.25 mm × 0.25 µm, or RXi-1MS (Restek, Bellefonte, PA, USA) 15 m × 0.25 mm × 0.25 µm capillary column. Before the analysis, the samples were diluted with ethanol by a factor of 10. The concentration of the obtained products in samples was evaluated based on the calibration curves for the known concentrations of external standards. The column oven temperature was programmed from 50 (5.5 min hold) to 290 °C (7.5 min hold) at 20 °C min^−1^. Helium was used as a carrier gas at a constant flow of 0.8 mL min^–1^. The temperature of the injector and detector was 290 °C, the injection volume has been set to 1 µL with a split ratio of 50. The separated compounds were identified using mass spectrometry. Every product was sent through the ion source and the fragments were separated by a single quadrupole in the range from 35 to 600 *m/z*. The mass spectra were compared to the spectra of pure compounds from commercial FFNSC and NIST17 libraries.

The quantitative GC–MS/FID data reported in this work are averages of three experiments. The maximum standard deviation of our results was 1.1 × 10^−3^ mol L^–1^, while the maximum standard error was 6.1 × 10^−4^ mol L^–1^.

### Lignin extraction

Lignin was extracted from beech tree sawdust (25 g, < 24 mesh, dried at 105 °C for 24 h) with methanol, ethanol, 75 vol% methanol/water and 75 vol% ethanol/water in the ratio of 1 : 7 (w/v). To catalyse the reaction, 1 % of H_2_SO_4_ based on dry matter was added to the reaction mixture. The extraction was carried out in a 300 mL cylindrical stainless steel slurry reactor (Autoclave Engineers) at 180 °C over 1 h. The reactor was flushed twice and pressurized with nitrogen up to 1 MPa. The experiments were performed in a batch regime with the agitation speed of 200 min^–1^. The reaction was quenched by dipping the reactor in an ice-bath. The solids were filtered out and rinsed with 150 mL of the 4 : 1 (m)ethanol/water mixture heated up to 60 °C in order to remove the extracted lignin, trapped on the surface of the wood particles. The remaining solids were dried at 105 °C for 48 h. Lignin was precipitated by adding three volumes of distilled water. The precipitate was collected by centrifugation for 10 min at 4,500 min^−1^, repeatedly washed with distilled water and freeze-dried. Yields of isolated lignin (%) were calculated according to Eq. 1 considering that beech wood contains 24.4 %^29^ of lignin.

1 $${\mathrm{Y}\mathrm{i}\mathrm{e}\mathrm{l}\mathrm{d} }_{Lignin}\left(\mathrm{\%}\right)= \left(\frac{{W}_{t }}{{W}_{0}}\right) \times 100$$

Here ${\mathrm{W}}_{t}$—weight of isolated lignin (g), ${\mathrm{W}}_{0}$—initial lignin content (g). The yield of residue (%) was calculated according to Eq. 2:

2 $${\mathrm{Y}\mathrm{i}\mathrm{e}\mathrm{l}\mathrm{d} }_{Residue}\left(\mathrm{\%}\right)= \left(\frac{{Z}_{t }}{{Z}_{0}}\right) \times 100$$

Here ${\mathrm{Z}}_{t}$—weight of the remaining solids (g), ${\mathrm{Z}}_{0}$—weight of starting beech wood (g).

### ATR/FT–IR spectroscopic analysis

Fourier Transform Infra–Red (FT–IR) spectra of isolated lignin samples were recorded using a FTIR spectrophotometer equipped with a LiTaO_3_ detector (PerkinElmer, FT–IR spectrophotometer, Spectrum Two, Manchester, UK), in the range between 400 and 4,000 cm^−1^ using diamond ATR mode of operation with 64 accumulated scans, at a resolution of 4 cm^−1^. The background spectrum was collected before every measurement and was subtracted from the sample spectrum automatically.

### Size-exclusion chromatography (SEC)

SEC of the acetylated lignin samples was performed on a size-exclusion chromatographic system (Thermo Scientific Ultimate 3000, ThermoFisher, Waltham, MA, USA) equipped with a UV detector set at 280 nm. The analyses were carried out at ambient temperature using THF as eluent at a flow rate of 1 cm^3^ min^−1^. The aliquots (100 µL) of each sample dissolved in THF (1.5 mg cm^−3^) were injected into Plgel 3 µm MIXED E 7.5 × 300 mm. The column specifications allow the separation by molecular weight up to 30 kDa. The SEC system was calibrated with polystyrene standards with the molecular weight in the range from 500 Da to 30 kDa. The chromatographic data were processed with the PSS (Polymer Standards Service) WinGPC Unity software. The acetylation of lignin was performed using acetic anhydride according to the procedure reported elsewhere^30^.

## Results and discussion

### Acidolysis of the α-O-4 linkage in non-aqueous alcohols

**Figure 1:**
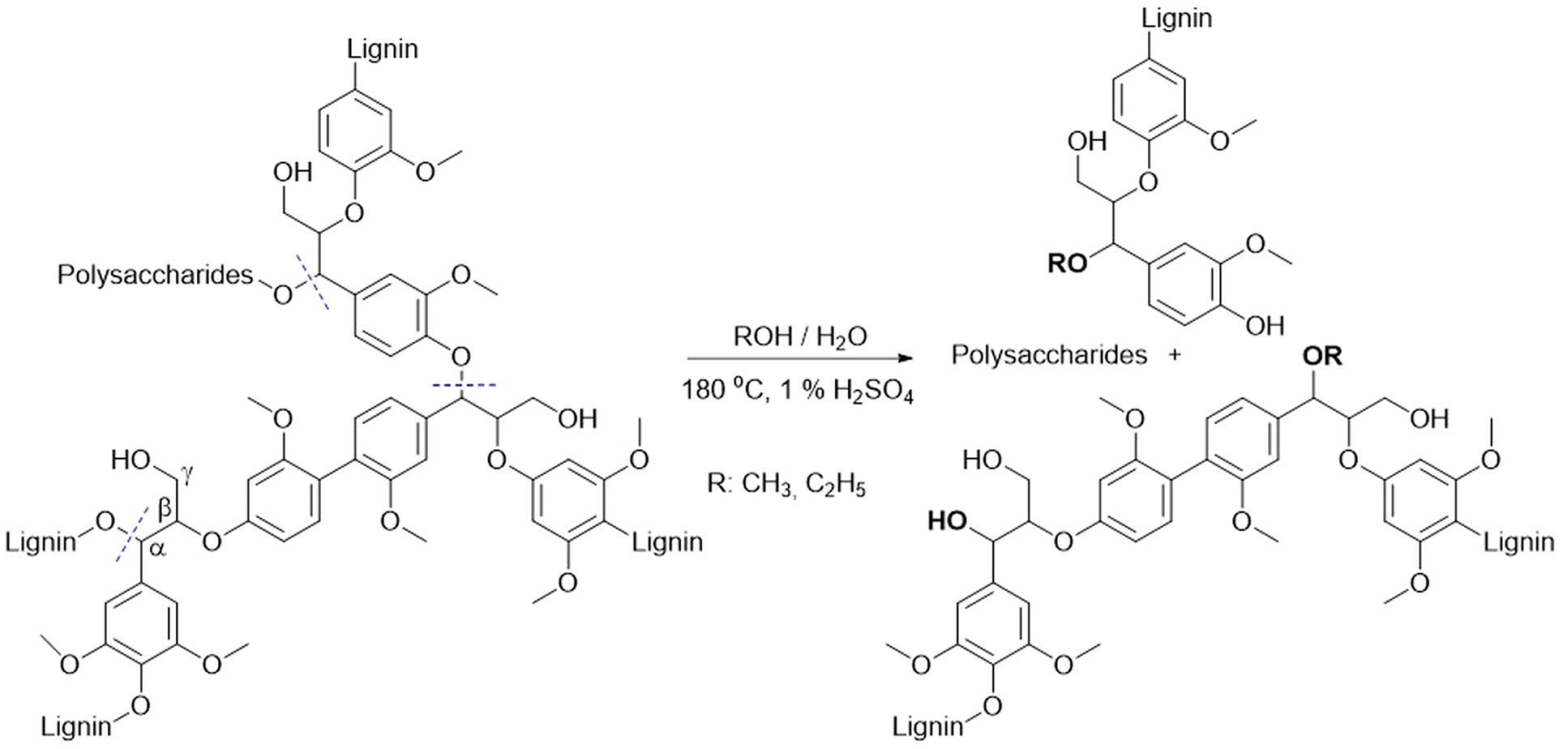
Cleavage of the α-ether bonds in lignin followed by alkoxylation during organosolv pretreatment.

It is well-known that during organosolv pretreatment lignin undergoes initial depolymerisation due to the scission of the α-ether bonds, which are cleaved more easily than the β-ether bonds because of a lower bond dissociation energy (BDE(α-O-4) = 215 kJ mol^–1^; BDE(β-O-4) = 290 kJ mol^–1^)^27^. Acidolysis of the α-ether linkage was found to predominate during the initial phase of organosolv pretreatment and was considered to be the rate-determining step. Subsequently, alkoxylation of a lignin macromolecule caused by alcohols acting as external nucleophiles determines the final structure of the isolated lignin as shown in Fig. 1^31^. Therefore, to elucidate the effect of the alcohol used, acidolysis of BPE (α-O-4 model compound) was examined in ethanol and methanol. Based on the GC–MS analyses, the main identified products were phenol (Ph), 2-benzyl phenol (2BPh) and benzyl ethyl ether (BEE) in the case of acidolysis in ethanol, and benzyl methyl ether (BME) in the case of acidolysis in methanol.

#### Reaction mechanism

The mechanism of acidolysis of benzyl phenyl ether (BPE) in the aqueous and non-aqueous alcohols was proposed based on the GC–MS analyses of the liquid samples. The main products of the BPE acidolysis in ethanol were identified to be phenol (Ph) and benzyl ethyl ether (BEE), while minor amounts of 2-benzyl phenol (2BPh) were also detected. Acidolysis in methanol proceeded in a similar manner, yielding benzyl methyl ether (BME) instead of BEE. The acid–catalysed conversion of BPE in ethanol with water as a co-solvent led to the formation of benzyl alcohol (BA). The product distribution was strongly affected by the solvent, acidity and temperature.

Ethers can be cleaved via the S_N_1 or S_N_2 mechanisms. We argue that BPE follows the S_N_1 mechanism due to the intermittent formation of a stable primary benzyl carbocation (stabilized by the resonance effect as presented in Supplementary Fig. S1) and due to the polarity of the medium. As shown below, increasing the polarity of the medium (substituting ethanol with methanol) increased the reaction rate.

**Figure 2:**
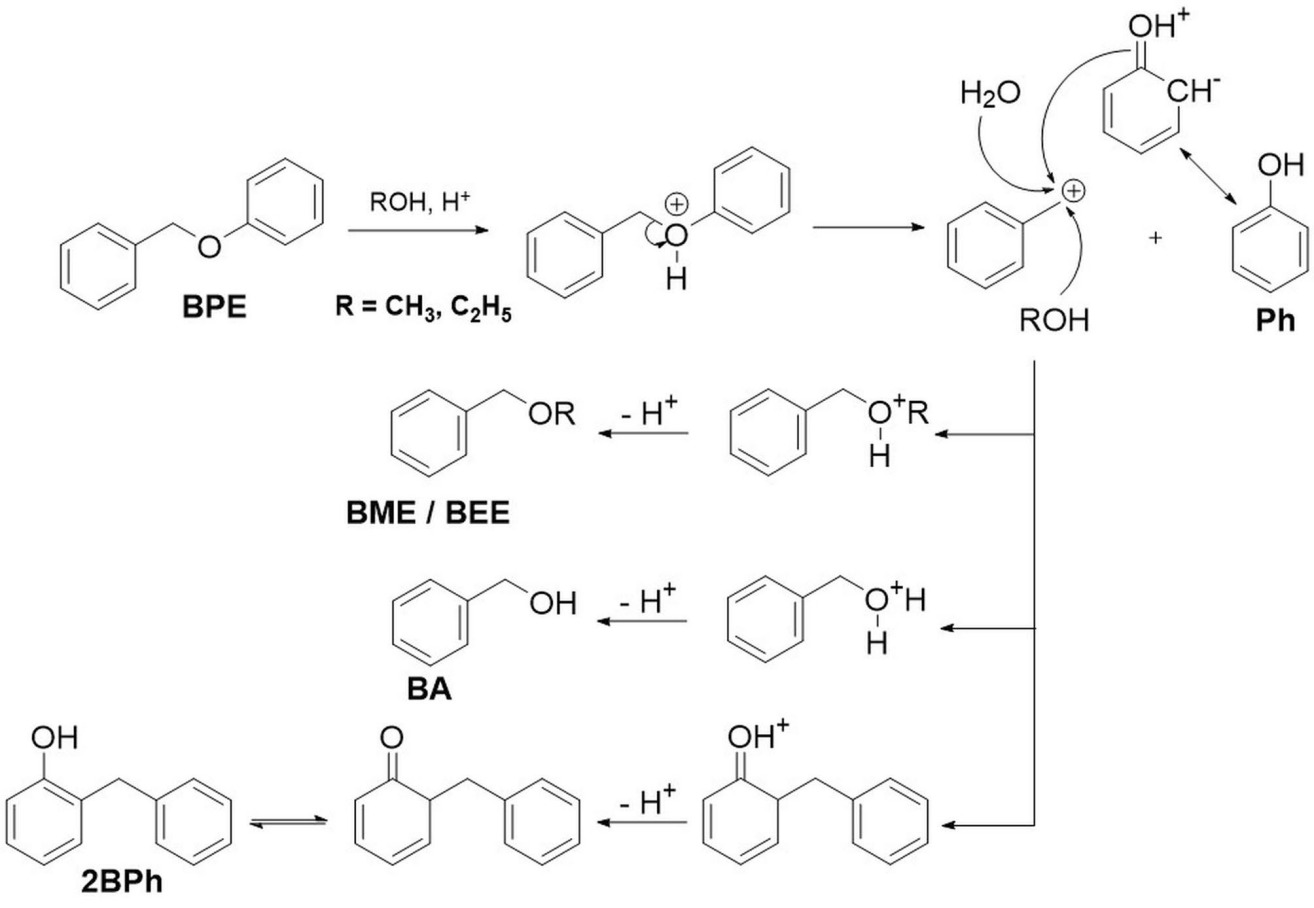
The proposed reaction mechanism of BPE acidolysis in aqueous and non-aqueous alcohol.

In Fig. 2, the mechanism of acidolysis is shown. In the first step, BPE is protonated by sulphuric acid. Subsequently, phenol and a primary benzylic carbocation are formed in a S_N_1 reaction. This carbocation reacts with either alcohol or water, forming BME/BEE or BA, respectively^32,33^.

Small quantities of 2BPh form when phenol reacts with the carbocation. If phenol reacts with its oxygen atom, this is a self-exchange reaction (the phenolic group in BPE is replaced by another phenol). However, the hydroxyl group in phenol activates the carbon atom in the *ortho* position, which can also take part in the acidolysis. This results in the formation of 2BPh (after keto-enol isomerization)^32,33,34^.

#### The effect of solvent

**Figure 3:**
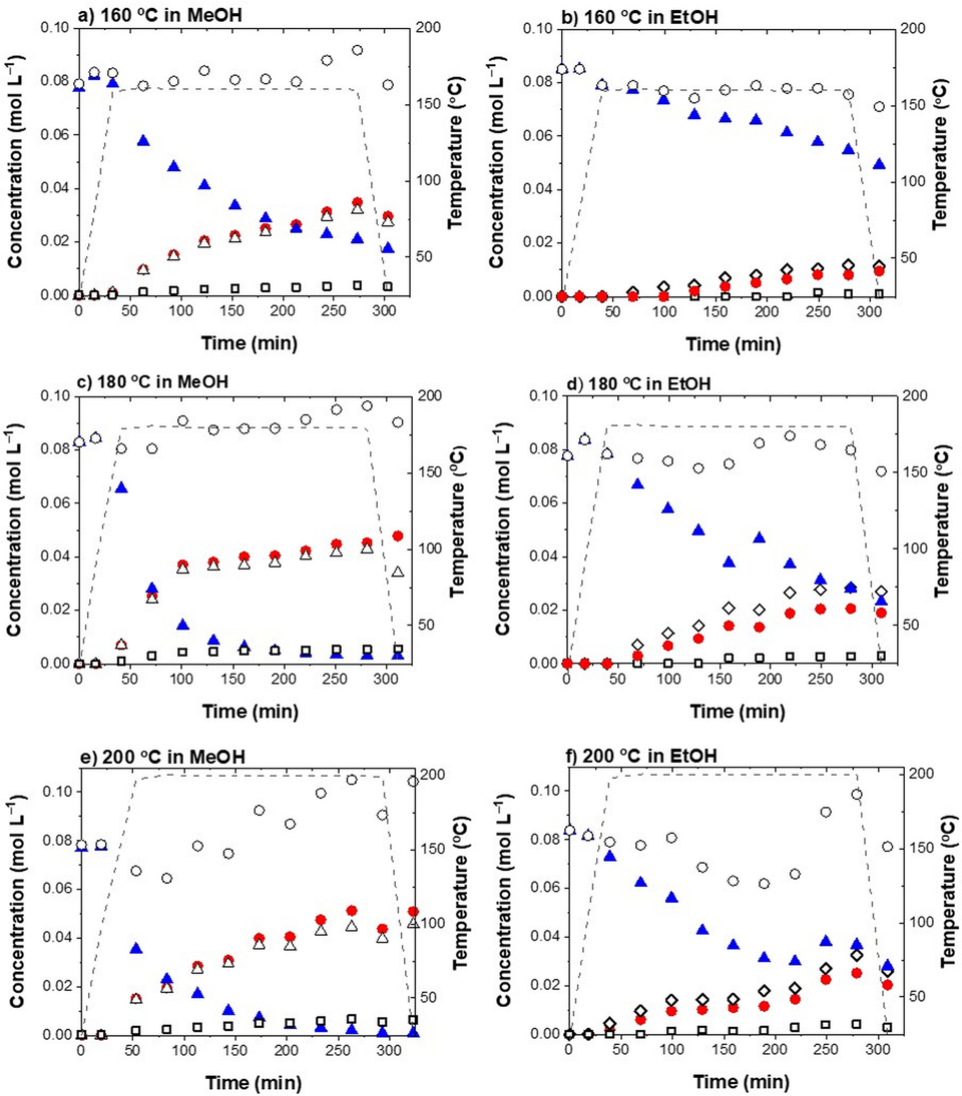
Product distribution during the BPE acidolysis in methanol (MeOH) or ethanol (EtOH) with 1.0 % of H_2_SO_4_ at 160 °C (**a**, **b**), 180 °C (**c**, **d**) and 200 °C (**e**, **f**). Key: *blue filled triangles* — benzyl phenyl ether (BPE), *black empty diamonds* — benzyl ethyl ether (BEE), *black empty triangles* — benzyl methyl ether (BME), *red filled circles* — phenol (Ph), *black empty squares* — 2-benzyl phenol (2BPh), *black empty circles* — molar balance.

The difference in the intensity of the alkoxylation reactions is most obvious when comparing the BPE, Ph, BME and BEE concentration profiles for the experiments performed with 1 % of H_2_SO_4_ at 160 °C (runs 10, 11), shown in Fig. 3a,b.

During the four-hour reaction, the BPE conversion of 77.5 % in methanol and 42.1 % in ethanol was achieved. We ascribe the greater activity in methanol to the difference in acidity *(methanol pK*_*a*_ = *15.5, ethanol pK*_*a*_ = *15.9)*^35^ and polarity (methanol > ethanol)^36^. Firstly, the O–H bond is more polarized in methanol (no stabilizing inductive effect), making it a better nucleophile to attack the benzyl carbocation. Moreover, greater polarity of methanol stabilizes the carbocation intermediate, which is well known to accelerate the S_N_1 reactions^32,33^.

#### The effect of temperature and acid concentration in methanol

**Figure 4:**
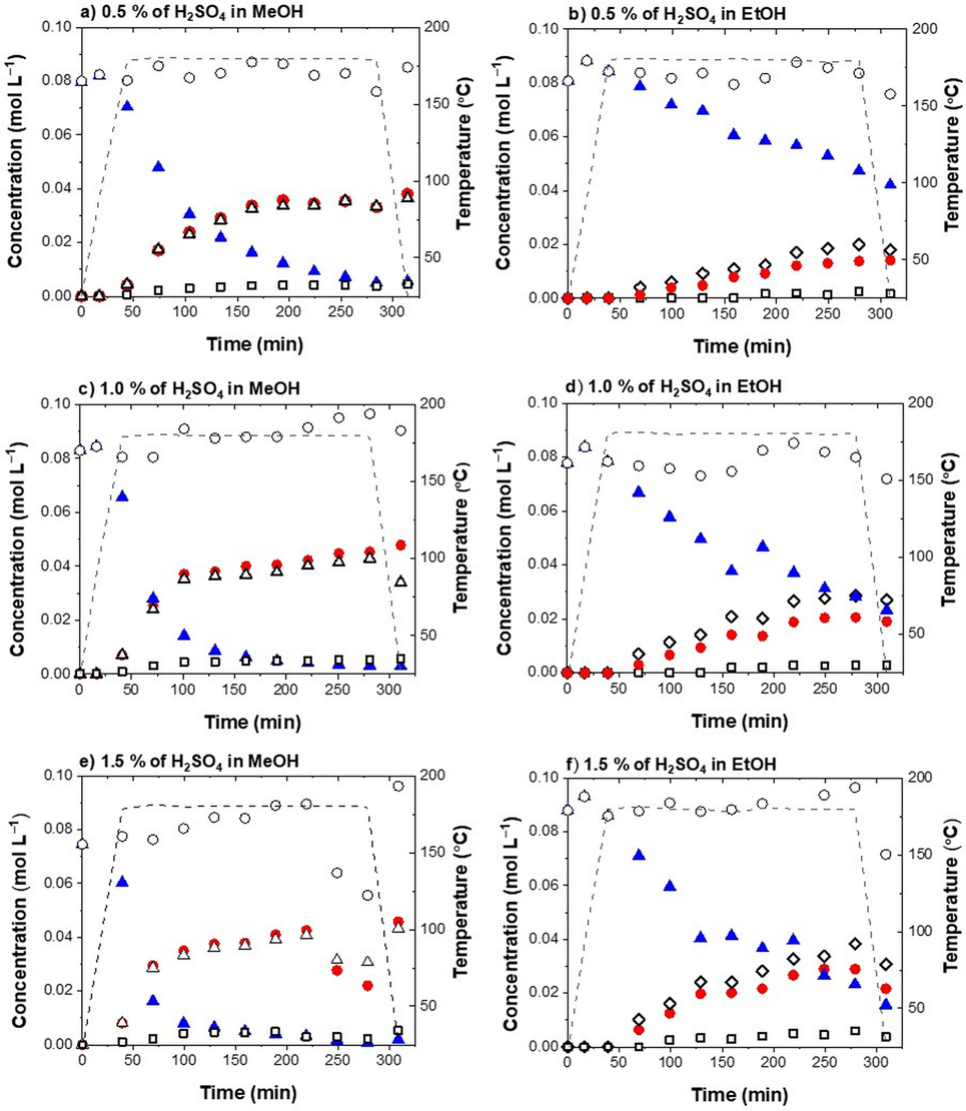
Product distribution during the BPE acidolysis in methanol (MeOH) or ethanol (EtOH) at 180 °C with 0.5 % of H_2_SO_4_ (**a**, **b**), 1.0 % of H_2_SO_4_, (**c**, **d**) and 1.5 % of H_2_SO_4_ (**e**, **f**). Key: *blue filled triangles* — benzyl phenyl ether (BPE), *empty diamonds* — benzyl ethyl ether (BEE), *empty triangles* — benzyl methyl ether (BME), *red filled circles* — phenol (Ph), *black empty squares* — 2-benzyl phenol (2BPh), *black empty circles*—molar balance.

To determine the effect of operating conditions, the experiments were performed in ethanol and methanol at different temperatures (160 °C, 180 °C, 200 °C; runs 10, 11, 4, 5, 13, 14) and with different catalyst concentrations (0.5 %, 1.0 %, 1.5 %; runs 1, 2, 4, 5, 7, 8), as shown in Figs. 3 and 4, respectively.

The concentration profiles of 2-benzyl phenyl (2BPh) are similar for all temperatures and both alcohols. However, slightly higher concentrations of 2BPh were observed in methanol. The more pronounced formation of 2BPh is the consequence of the overall greater reactivity in methanol.

**Figure 5:**
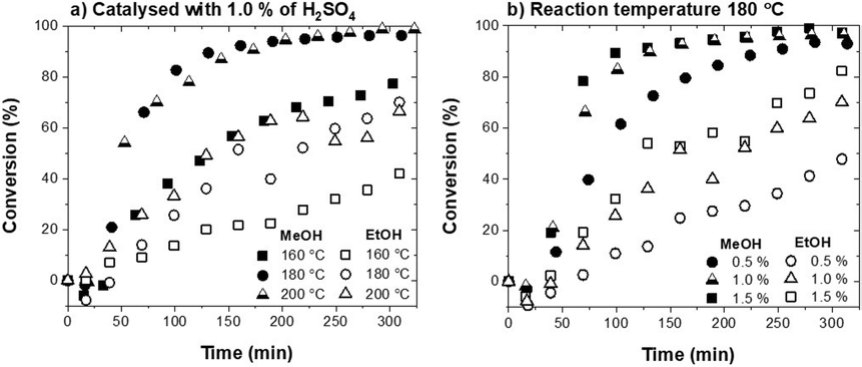
Benzyl phenyl ether (BPE) conversion with time in methanol (MeOH) and ethanol (EtOH): (**a**) at 160 °C, 180 °C, 200 °C with 1.0 % of H_2_SO_4_ used as a catalyst; (**b**) at 180 °C with 0.5 %, 1.0 % and 1.5 % of H_2_SO_4_.

The influence of temperature on the BPE conversion is presented in Fig. 5a, while Fig. 5b shows the effect of acid concentration. The temperature effect is most obvious. Increasing the temperature from 160 to 200 °C increased the conversion in methanol from 77.5 to 98.8 % and in ethanol from 42.1 to 70.0 %. With methanol, the conversion was nearly complete (96.4 %) already at 180 °C and only negligible improvement was observed with a further increase of the reaction temperature to 200 °C. A similar effect was seen when using ethanol, only the conversions were not as high (vide infra).

The concentration of acid had a small effect on the conversion. Upon comparing the experiments at 180 °C with 0.5 %, 1.0 % and 1.5 % of H_2_SO_4_ (Fig. 4a,c,e), no noticeable difference in the conversion is seen (93 %, 96.4 % and 97.4 %, respectively).

The effect on the initial reaction rate was more distinct, however not directly proportional to the amount of the used catalyst. For instance, 70 % of the BPE conversion during the acidolysis in methanol at 180 °C acidified with 0.5 %, 1.0 % and 1.5 % of H_2_SO_4_ was achieved after 130 min, 75 min and 55 min, respectively (Fig. 5b). Based on the obtained results, we assume that the alcohol acidity (pK_a_) and polarity are the key parameter, determining the intensity of the α-ether bond acidolysis in non-aqueous alcohols. The rate of ether bond acidolysis is more strongly dependent on the reaction temperature than on the concentration of hydronium ions.

#### The effect of temperature and acid concentration in ethanol

A similar temperature effect as in methanol has been observed also in ethanol, acidified with 1.0 % of H_2_SO_4_ (Fig. 3b,d,f). Here, increasing the reaction temperature from 160 to 180 °C caused an analogous increase in the α-ether bond cleavage. After four hours of the reaction, the conversion rose from 42.1 to 70.0 %. Analogously, any further rise of the reaction temperature (up to 200 °C) had a negligible effect on the conversion. This suggests that the optimal temperature for the cleavage of the α-O-4 linkage is 180 °C.

The product distribution in ethanol was significantly affected by the acidity of the reaction media. As shown in the concentration profiles in Fig. 4b,d,f, the effect of the acid concentration in ethanol has a much more pronounced effect than in the case of methanol. When running the reaction in ethanol with 0.5 %, 1.0 % and 1.5 % of H_2_SO_4_ at 180 °C, the conversions were 47.9 %, 70.13 % and 82.3 %, respectively. This is a consequence of the different polarity of methanol and ethanol, as discussed (vide supra)^36^.

From the obtained results we conclude that the *extent* and *rate* of the α-O-4 bond cleavage is strongly dependent on the type of the solvent, specifically the acidity and polarity of the solvent itself. A greater solvent polarity can be beneficial as it reduces the required amount of sulfuric acid, which is the most frequently used catalyst for organosolv pretreatment. Expectedly, the reaction is also influenced by the concentration of the hydronium ion (a catalyst) and temperature.

### Acidolysis of the α-O-4 linkage in aqueous ethanol

#### The effect of water

Water is the most environmentally friendly solvent and is usually used in combination with organic solvents for organosolv pretreatment^37,38^. The highest lignin solubility in ethanol–water mixtures has been achieved in 70 % ethanol/water^39^. According to the Hildebrand solubility parameter theory, the materials having similar δ-values show good solubility or miscibility. It has been determined that good solvents for lignin have the δ-value around 11 (cal cm^–3^)^1/2^^40^. An efficient dissolution of high and low molecular weight lignin fractions has been demonstrated in a mixture of 75 % ethanol (δ = 12.9 (cal cm^–3^)^1/2^) and 25 % of water (δ = 23.5 (cal cm^–3^)^1/2^) with the δ-value close to the one of lignin (δ = 12 − 15.5 (cal cm^−3^)^1/2^)^41,42,43^.

Hence, 75 vol% ethanol/water was examined as a solvent in our study. We sought to determine the effect of water on the α-ether bond acidolysis. Additionally, the influence of the reaction temperature and acidity of the reaction media was investigated. A *methanol*/water mixture was not studied due to poor solubility of the model compound (BPE).

Based on the GC–MS analyses, the main identified products were phenol (Ph), 2-benzyl phenol (2BPh), benzyl ethyl ether (BEE) and benzylic alcohol (BA). As shown in Fig. 2, water or ethanol participate in the S_N_1 reaction after the protonation step, yielding BA or BEE, respectively.

**Figure 6:**
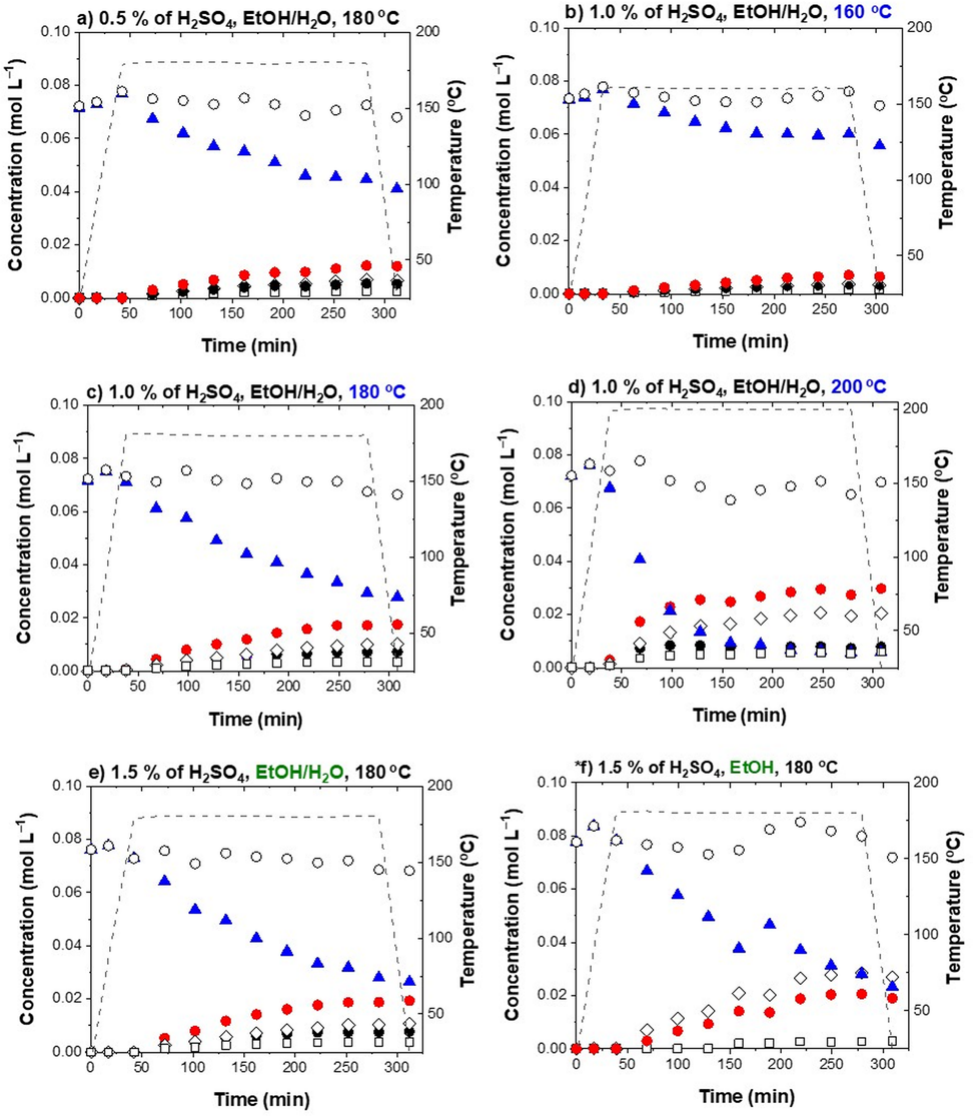
Product distribution during BPE acidolysis at 180 °C in ethanol/water (EtOH/H_2_O) with (**a**) 0.5 % of H_2_SO_4_, (**c**) 1.0 % of H_2_SO_4_, (**e**) 1.5 % of H_2_SO_4_. The temperature influence on the product distribution in EtOH/H_2_O with 1.0 % of H_2_SO_4_ at: (**b**) 160 °C, (**c**) 180 °C, (**d**) 200 °C. *(**f**) The product distribution in EtOH with 1.5 % of H_2_SO_4_ at 180 °C is shown again for a direct comparison with the effect of water. Key: *blue filled triangles* — benzyl phenyl ether (BPE), *empty diamonds* — benzyl ethyl ether (BEE), *black filled circles* — benzyl alcohol (BA), *red circles* — phenol (Ph), *black empty squares* — 2-benzyl phenol (2BPh), *black empty circles* — molar balance.

The intensity of the reaction is most evident in the BPE, Ph, BEE and BA concentration profiles for the experiments performed with 1.5 % of H_2_SO_4_ at 180 °C (runs 7, 9), shown in Fig. 6e and f. After four hours, the conversion was 82.3 % in ethanol and 65.3 % in 75 % ethanol/water was achieved.

The lower activity in the ethanol/water mixture compared to ethanol might be explained by its acidity. Water itself (pK_a_ =  − log (K_w_/(55.56 mol L^–1^)) = 15.7) is *more* acidic than ethanol (pK_a_ = 15.9)^35^. This means that H_2_SO_4_ more easily protonates ethanol than water, causing the reaction to be slower in the ethanol/water mixture.

However, one of the main challenges is an efficient lignin isolation with as few structural changes (condensation reactions, scission of β-ether linkages and alkoxylation reactions) as possible. During organosolv pretreatment it is important to preserve the reactive functional groups (aliphatic and phenolic hydroxyl groups). We show that the intensity of the ethoxylation reactions can be reduced by a factor of 3 by adding 25 % of water to ethanol. Water evidently represents one of the key parameters and has to be taken into account for eventual lignin isolation by the organosolv fractionation.

#### The effect of temperature

The effect of temperature on the α-ether scission in acidified aqueous ethanol was tested at 160 °C, 180 °C and 200 °C (runs 12, 6, 15) with 1.0 % of H_2_SO_4_. The product distribution in aqueous ethanol at different temperatures is shown in Fig. 6.

The effect is most evident in the concentration profiles of BPE. As the temperature rises from 160 to 200 °C, α-ether bond cleavage is significantly promoted and the conversion increases from 23.7 to 92.0 %. The temperature increase significantly increased the reaction *rate* and strongly affected the product distribution.

The reactant conversion in aqueous ethanol as a function of the reaction time is shown in Supplementary Fig. S2. We see that a 15 % conversion of BPE in aqueous ethanol with 1 % of H_2_SO_4_ at 160 °C, 180 °C, 200 °C was achieved after 150 min, 75 min, 50 min, respectively. The highest catalyst activity and conversion (up to 92.0%) was demonstrated at 200 °C with 1.0 % of H_2_SO_4_, yielding the highest concentrations of Ph, BEE and BA. Moreover, the BEE : BA ratio at the end of the experiment was 3 : 1, which corresponds to the initial ratio of ethanol and water. This additionally confirms the S_N_1 reaction mechanism shown in Fig. 2.

The concentration profiles of 2BPh exhibit similar patterns. For instance, the concentrations of 2BPh determined after a four-hour treatment with 1.0 % of H_2_SO_4_ at 160 °C, 180 °C, 200 °C were proportional to the Ph content and made up approximately 20 % of phenol.

#### The effect of acidity

To evaluate the effect of acidity on the α-ether bond scission in aqueous ethanol, experiments were carried out at 180 °C with 0.5 %, 1.0 % and 1.5 % of H_2_SO_4_ (runs 3, 6, 9). The product distributions during the reaction are shown in Fig. 6. The BPE concentration profiles show that an increase in acidity (from 0.5 to 1.5 %) accordingly affects the α-ether bond cleavage, as evidenced by an increase in the conversion (from 42.1 to 65.3 %).

The conversion of the reactant in aqueous ethanol as a function of time is shown in Supplementary Fig. S2. For instance, a 30 % conversion in aqueous ethanol with 0.5 %, 1.0 % and 1.5 % of H_2_SO_4_ at 180 °C was achieved after 200 min, 125 min and 100 min, respectively. This shows that after a certain threshold (in our case 1.0%), the increased acidity does not translate into higher reaction rate.

However, when carrying out the reaction in ethanol, the effect was more pronounced. Increasing the concentration of H_2_SO_4_ from 0.5 to 1.5 % improved the final conversion from 47.9 to 82.3 %. This is consistent with the overall *decrease* of the reaction rate when water is added to ethanol. A greater amount of the catalyst is required, meaning that the threshold is moved above 1.0 %. Due to the higher acidity of water compared to ethanol, H_2_SO_4_ is less likely to dissociate and protonate it, results in the overall lower concentration of protonated species (hydronium ions or alcohols).

Overall, the addition of water has a desired effect on the reaction. Firstly, the addition of 25 % of water to ethanol decreased the extent of alkoxylation reaction by a factor of 3, preserving the reactive hydroxyl group. Specifically, concentration of benzyl ethyl ether (BEE) has been reduced from 0.03 to 0.01 mol L^−1^ (Figs. 3d and 6c). Secondly, a more pronounced temperature-acidity dependence in aqueous ethanol is a key parameter to be considered for attaining the optimal extent of α-ether bond cleavage. Thirdly, by comparing the patterns in the concentration profiles in Figs. 3 and 4 with Fig. 6, the addition of water has reduced experimental variations, resulting in a more consistent process.

### The effect of solvent on lignin

To estimate the effect of solvents tested with the model compound BPE, lignin was isolated from beech wood with MeOH, EtOH, MeOH/H_2_O, EtOH/H_2_O under moderate conditions at 180 °C with 1.0 % of H_2_SO_4_ according to the procedure described in Section “Material and methods.”

#### ATR/FT-IR spectroscopic analysis

**Figure 7:**
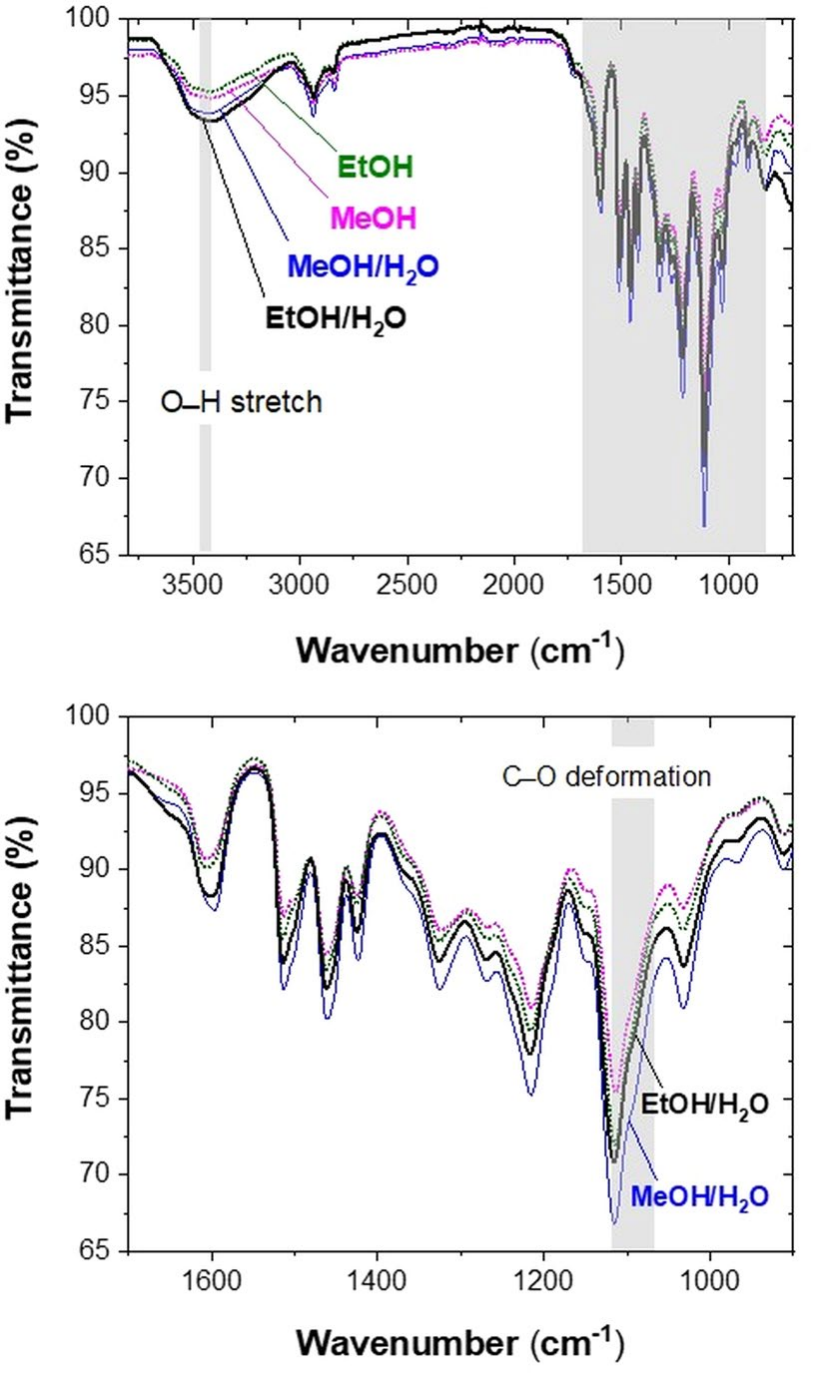
ATR-FTIR spectra of lignin isolated in MeOH, EtOH, MeOH/H_2_O and EtOH/H_2_O with the enlarged highlighted area of the original spectra.

Preliminary information on the amount of the total hydroxyl groups was obtained using a FTIR analysis. The FTIR spectra were collected in ATR mode, which enables a simple and fast comparison of lignin samples. The assignment of lignin FTIR spectra was reported in detail by Faix^44^ and allows the identification of characteristic bands for total hydroxyl groups (O–H stretch) at 3,412–3,460 cm^–1^ as well as for secondary alcohols and aliphatic ethers (C–O deformation) at 1,086 cm^–1^. Figure 7 shows the FTIR spectra of lignin isolated with four different solvents (separate spectrum for each lignin sample are shown in Supplementary Figs. S3–S6).

Intensity of the absorption band corresponding to the total OH groups increases in the following sequence: EtOH < MeOH < MeOH/H_2_O < EtOH/H_2_O and thus confirms that the addition of water to alcohol reduces the alkoxylation reactions preserving more OH groups in lignin. While there are minor differences between the spectra of lignin isolated using non-aqueous alcohols, the addition of water into the system causes observable changes. Lignin, isolated in aqueous methanol, exhibits a lower absorption band intensity corresponding to the O–H stretch and consequently a lower OH group content compared to the one isolated in aqueous ethanol. This difference could be explained by the different acidity of the solvents. As already mentioned, acidity increases in the following order EtOH (*pK*_*a*_ = *15.9*) < H_2_O (*pK*_*a*_ = *15.7*) < MeOH (*pK*_*a*_ = *15.5*)^35^. When aqueous methanol is used for lignin isolation, H_2_SO_4_ more easily protonates methanol than water, affecting the predominance of the transetherification reactions at the Cα position (alkoxylation). Moreover, the aliphatic α-ether formation is confirmed by a notably more intensive shoulder at 1,086 cm^–1^ compared to the one of lignin isolated in aqueous ethanol. In contrast to the aqueous methanol, the easier protonation of water than ethanol in aqueous ethanol, makes the α-ether bond hydrolysis the preferential reaction resulting in the formation of the aliphatic OH groups at the Cα position and accordingly increases overall OH group content. Correspondingly, the FTIR spectrum of lignin isolated in aqueous ethanol displays the highest band intensity corresponding to the O–H stretch (3,412–3,460 cm^–1^) along with the less intensive shoulder at 1,086 cm^–1^ revealing a reduced extent of the transetherification of α-ethers (alkoxylation).

#### Fractionation

The beech wood fractionation results are summarized in Supplementary Table S2. The highest yields of lignin were obtained using aqueous alcohols. Despite the comparable yields of residue, significant differences between the lignin yields (76.0 % and 83.4 %) were achieved using MeOH/H_2_O and EtOH/H_2_O, respectively. As expected, EtOH/H_2_O showed the most desired results. The residue obtained after the fractionation in MeOH/H_2_O had a notably darker colour and apparently a certain amount of lignin remained trapped on the surface of the particles. MeOH/H_2_O seems to be a less efficient solvent for lignin which is also in agreement with the Hildebrand solubility parameter theory, were the lignin (δ = 12 – 15.5 (cal cm^–3^)^1/2^) solubility in EtOH/H_2_O (δ = 15.5 (cal cm^–3^)^1/2^) should be better compared to MeOH/H_2_O (δ = 16.6 (cal cm^−3^)^1/2^)^41,42,43^. In addition, a higher degree of methylation in aqueous methanol limited a sufficient lignin isolation by precipitation in water and in the end, a yield of 76 % was obtained. The effect of more pronounced (m)ethylation is evident in the case of fractionation performed using MeOH and EtOH. In those two cases, the precipitated lignin formed a milky suspension that made its separation extremely complicated. Therefore, despite the beneficial δ-values that describe MeOH (δ = 14.3 (cal cm^–3^)^1/2^) and EtOH (δ = 12.9 (cal cm^–3^)^1/2^) as suitable solvents, notably lower lignin yields (70.6 % and 64.4 %, respectively) were attained.

The different degree of lignin (m)ethylation which is also confirmed using FTIR analysis, accordingly affected lignin self-aggregation intensity. Self-aggregation of the dissolved lignin depends on the balance of electrostatic repulsion and van der Waals attraction in a solvent as well as on the isolation process which directly affects the number of cross-linking sites (methoxyl and hydroxyl groups)^45^. Accordingly, due to the relatively low hydroxyl group content in lignin isolated using EtOH and MeOH formed a milky suspension during the precipitation. While the higher degree of lignin methylation in MeOH/H_2_O than the degree of ethylation in EtOH/H_2_O was confirmed by the notably reduced lignin self-aggregation intensity.

#### SEC analysis

**Figure 8:**
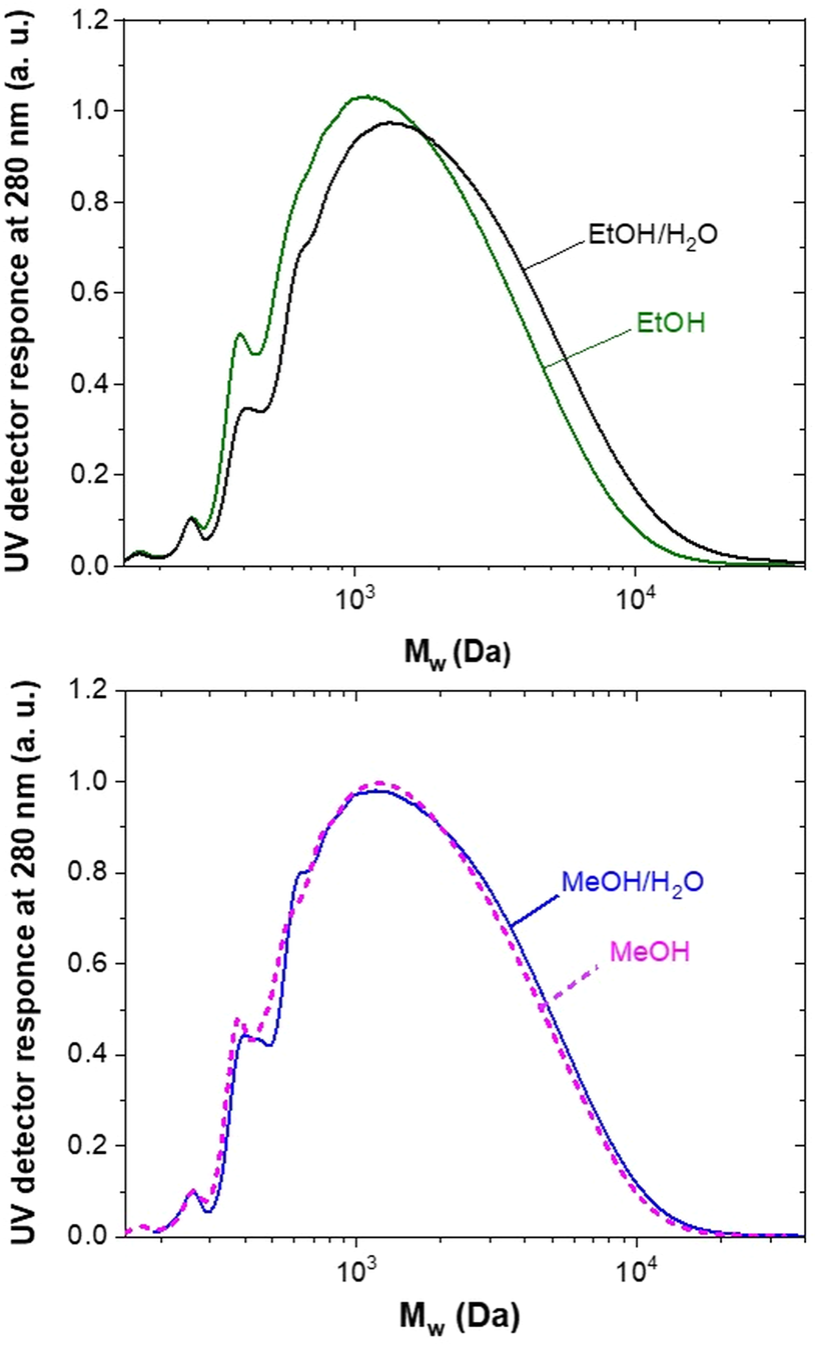
SEC chromatograms of lignin isolated using EtOH, EtOH/H_2_O, MeOH and MeOH/H_2_O.

The effect of the used solvents is also evident from the SEC analysis data shown in Supplementary Table S2. The formation of α-etherified moieties within the lignin structure during the fractionation in MeOH and EtOH prevents any further condensation reactions resulting lignin with a lower average molecular weight (M_w_) 2,150 Da and 2,020 Da, respectively. The presence of water in MeOH/H_2_O and EtOH/H_2_O creates reactive secondary OH groups that could be further etherified or involved in condensation with the other lignin functional groups, forming slightly larger fragments with an average M_w_ of 2,300 Da and 2,700 Da, respectively. However, based on the results of the previously investigated BPE acidolysis in EtOH/H_2_O, the presence of water is more likely to reduce the rate of the reaction or lignin depolymerisation in this case. Thus, higher M_w_ values for the lignin isolated in aqueous alcohols imply a lower degree of depolymerisation. The effect of water in terms of weaker depolymerisation is obvious from the SEC chromatogram profiles shown in Fig. 8. Here, the main peak of EtOH/H_2_O-lignin is shifted towards the higher molecular weight region pointing towards a presence of a less intact lignin structure. The minor difference between the average M_w_ values of MeOH-lignin (2,150 Da) and MeOH/H_2_O-lignin (2,300 Da) could be explained by the acidity of the used solvents and is consistent with the previously discussed results from the FTIR analyses. When MeOH/H_2_O is used for lignin isolation, H_2_SO_4_ more easily protonates methanol than water, affecting the predominance of the α-etherification reactions. Consequently, as a majority of the solvent mixture consists of MeOH (75 vol%), only a minor difference between the M_w_ values has been observed.

## Conclusions

In this study, benzyl phenyl ether (BPE) was used as a model compound for the α-O-4 ether linkage in lignin to understand the intricacies of the α-ether bond cleavage in acidified methanol, ethanol and in aqueous ethanol.

When using BPE, the S_N_1 mechanism was postulated based on several findings. Firstly, the primary benzyl carbocation is sufficiently stable to intermittently form after the protonation of BPE. Secondly, the reaction rate was found to be accelerated in methanol, which is more polar than ethanol, thus stabilizing the carbocation. Thirdly, when using the ethanol/water mixture, the ratio of the corresponding products (BEE:BA) matched the ratio of ethanol and water in the solvent, meaning that they compete for the carbocation.

The product distribution in non-aqueous alcohols was strongly affected by the solvent acidity and polarity, and temperature while the acidity of the reaction media had a less significant effect. Adding water to ethanol had a beneficial effect on the α-ether bond cleavage, especially in terms of alkoxylation reactions. Specifically, the extent of alkoxylation was reduced by a factor of 3, thus preserving the reactive hydroxyl group.

The structural differences between the lignins, isolated with (aqueous) alcohols, were consistent with the results obtained from the BPE acidolysis. Specifically, a reduced extent of the alkoxylation reactions reflected in a less depolymerized lignin molecule thus specifying 75 vol% EtOH/H_2_O as the most favourable solvent among the ones considered in this study.

## Supplementary information

Supplementary information
