# Supplementary material for: Acid-catalysed α-O-4 aryl-ether bond cleavage in methanol/(aqueous) ethanol: understanding depolymerisation of a lignin model compound during organosolv pretreatment
Source: Sci Rep. 2020 Jul 6;10:11037. doi: 10.1038/s41598-020-67787-9 (PMC7338503; doi:10.1038/s41598-020-67787-9)
Supplement: Supplementary file 1 — Supplementary information [file 41598_2020_67787_MOESM1_ESM.pdf]

ACID-CATALYSED α-O-4 ARYL-ETHER BONDS CLEAVAGE IN METHANOL AND (AQUEOUS) ETHANOL: DEPOLYMERISATION OF A LIGNIN MODEL COMPOUND DURING ORGANOSOLV PULPING

Edita Jasiukaitytė-Grojzdek, Matej Huš, Miha Grilc*, Blaž Likozar*

Department of Catalysis and Chemical Reaction Engineering, National Institute of Chemistry Hajdrihova 19, SI-1000 Ljubljana, Slovenia

Material and methods

Acidolysis experiments

Table S1. A list of the acidolysis experiments with BPE in ethanol, methanol or a 75 vol% ethanol/water mixture. The experiments were performed under an inert atmosphere of nitrogen at 1 MPa with H_2SO_4 as a catalyst.

Exp:	Solvent	H_2SO_4 [%][a]	*T* [°C]
1	EtOH	0.5	180
2	MeOH	0.5	180
3	EtOH/H_2O	0.5	180
4	EtOH	1.0	180
5	MeOH	1.0	180
6	EtOH/H_2O	1.0	180
7	EtOH	1.5	180
8	MeOH	1.5	180
9	EtOH/H_2O	1.5	180
10	EtOH	1.0	160
11	MeOH	1.0	160
12	EtOH/H_2O	1.0	160
13	EtOH	1.0	200
14	MeOH	1.0	200
15	EtOH/H_2O	1.0	200
16	EtOH	0	200
17	MeOH	0	200

[a] for 1 g of model compound 0.025 – 0.075 g of H_2SO_4 in form of 2M solution were used, thus taking into account that wood contains approximately 20 % of lignin

*Corresponding authors, Tel.: +386 1 4760 540; Fax: +386 1 4760 300; E-mail address: miha.grilc@ki.si; blaz.likozar@ki.si

Results and Discussion

Reaction mechanism

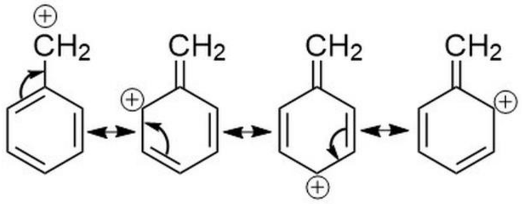

Figure S1. Stabilization of the benzyl carbocation by the resonance effect.

The effect of temperature

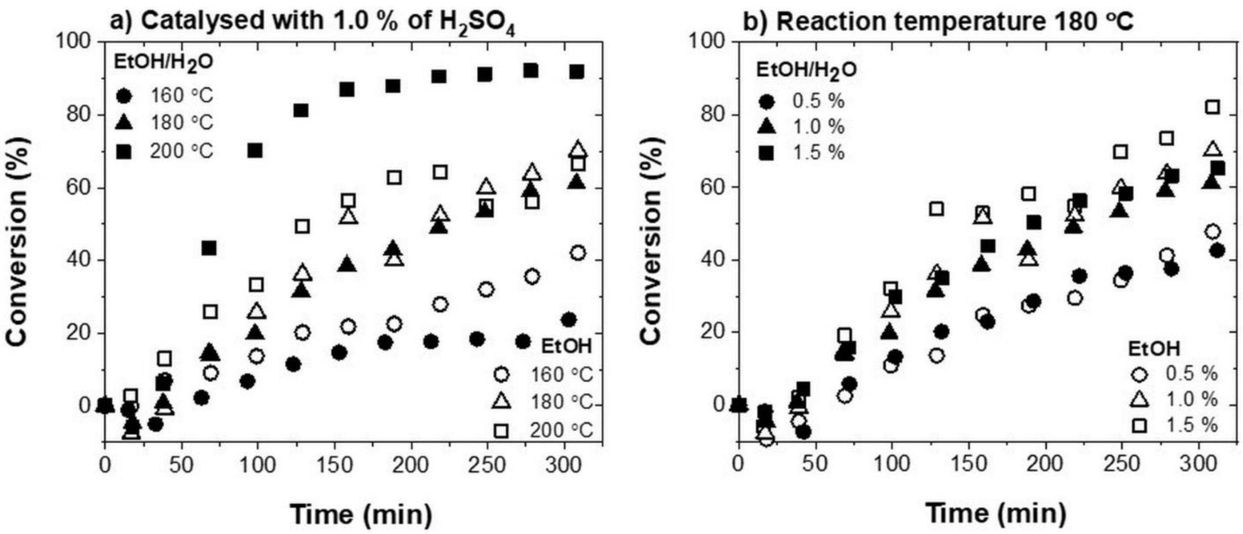

Figure S2. Conversion of benzyl phenyl ether (BPE) as a function of time in ethanol (EtOH)* and 75 vol% ethanol/water (EtOH/H_2O): a) at 160 ºC, 180 ºC, 200 ºC with 1 % of H_2SO_4. b) at 180 ºC with 0.5 %, 1.5 % and 1.5 % of H_2SO_4; *BPE conversions in EtOH are shown again for easier comparison.

ATR/FT-IR spectroscopic analysis

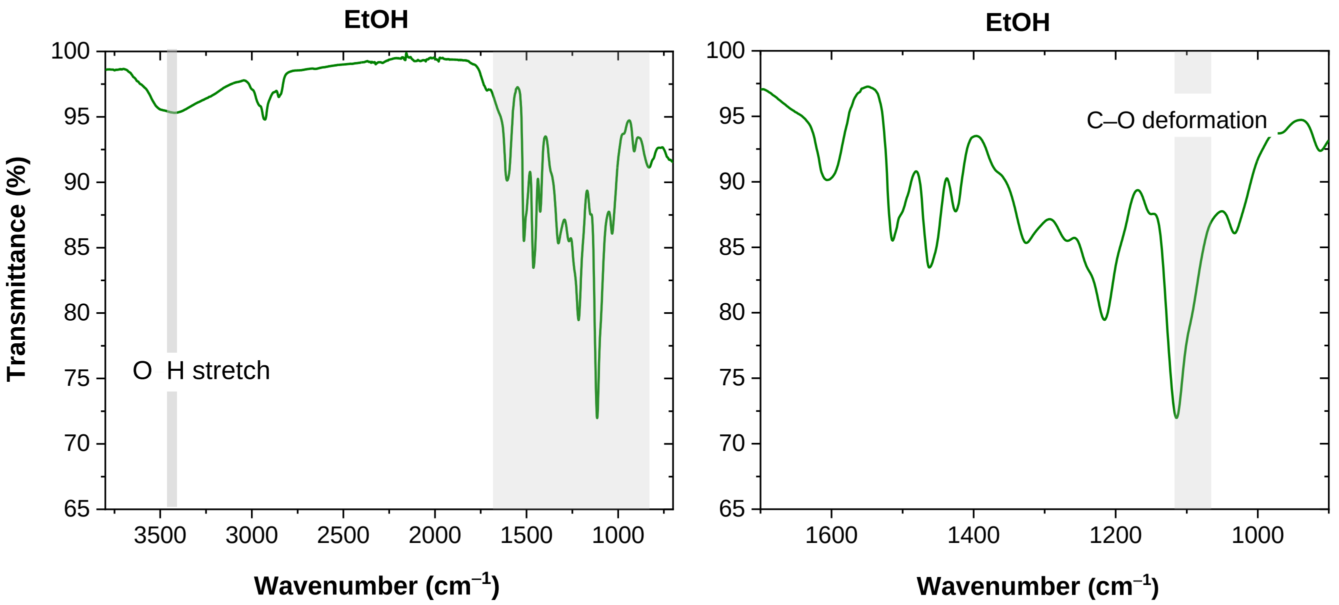

Figure S3. ATR-FTIR spectra of lignin isolated in EtOH with the enlarged highlighted area of the original spectra.

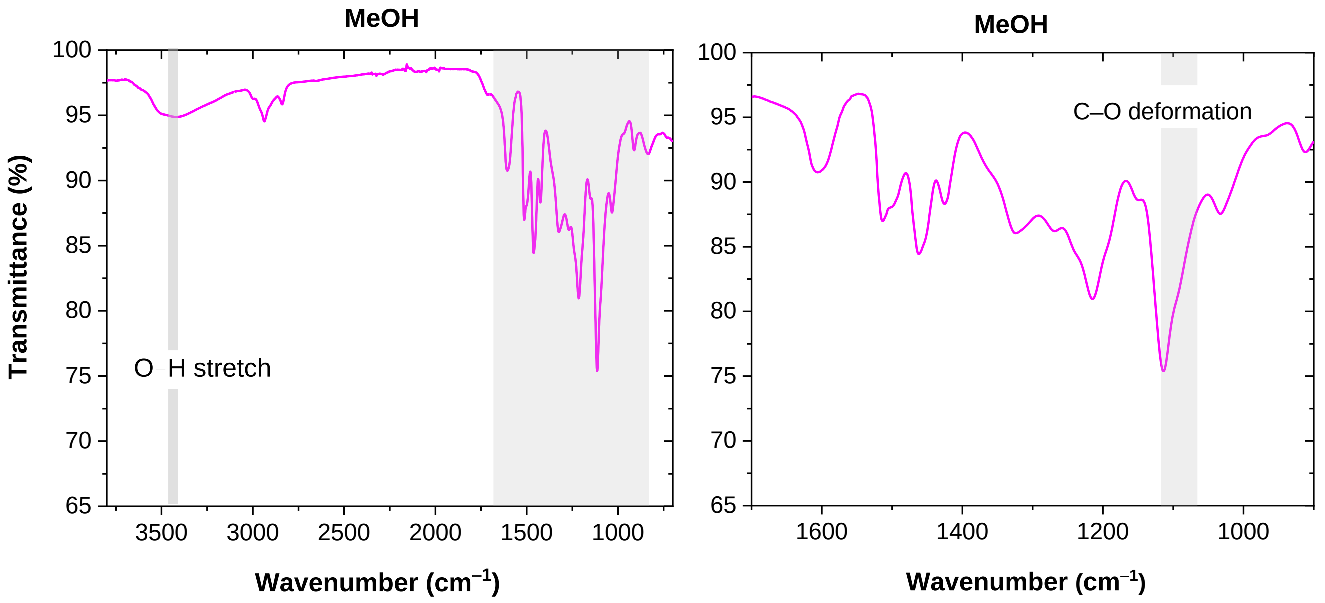

Figure S4. ATR-FTIR spectra of lignin isolated in MeOH with the enlarged highlighted area of the original spectra.

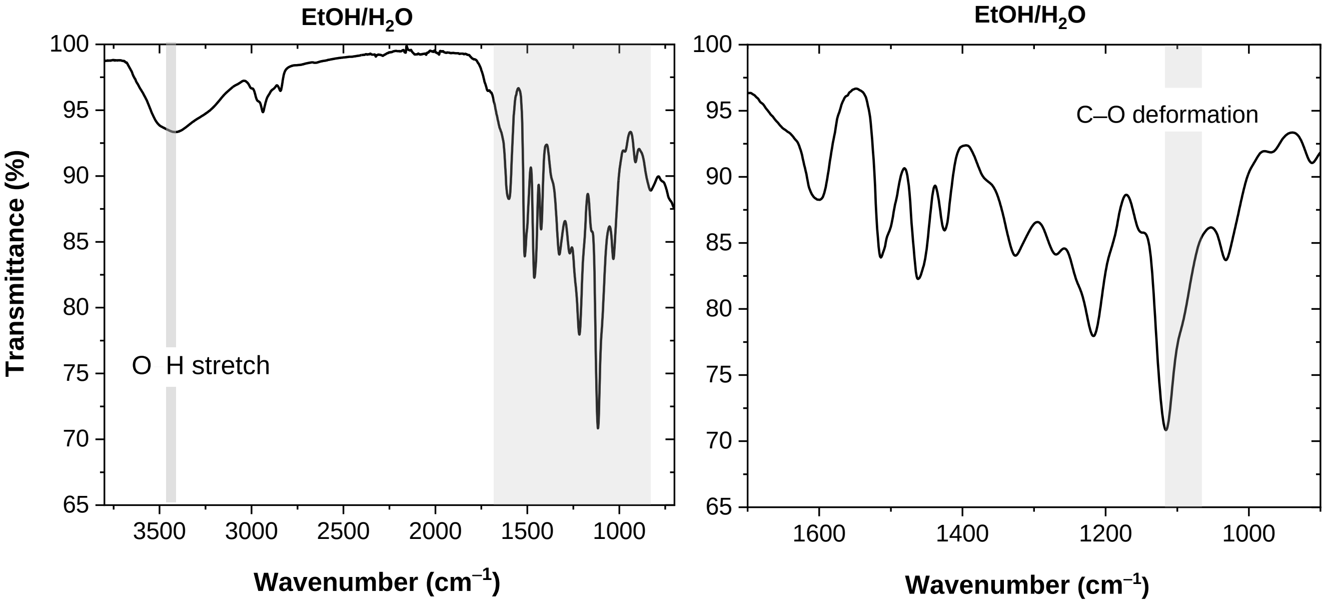

Figure S5. ATR-FTIR spectra of lignin isolated in EtOH/H_2O with the enlarged highlighted area of the original spectra.

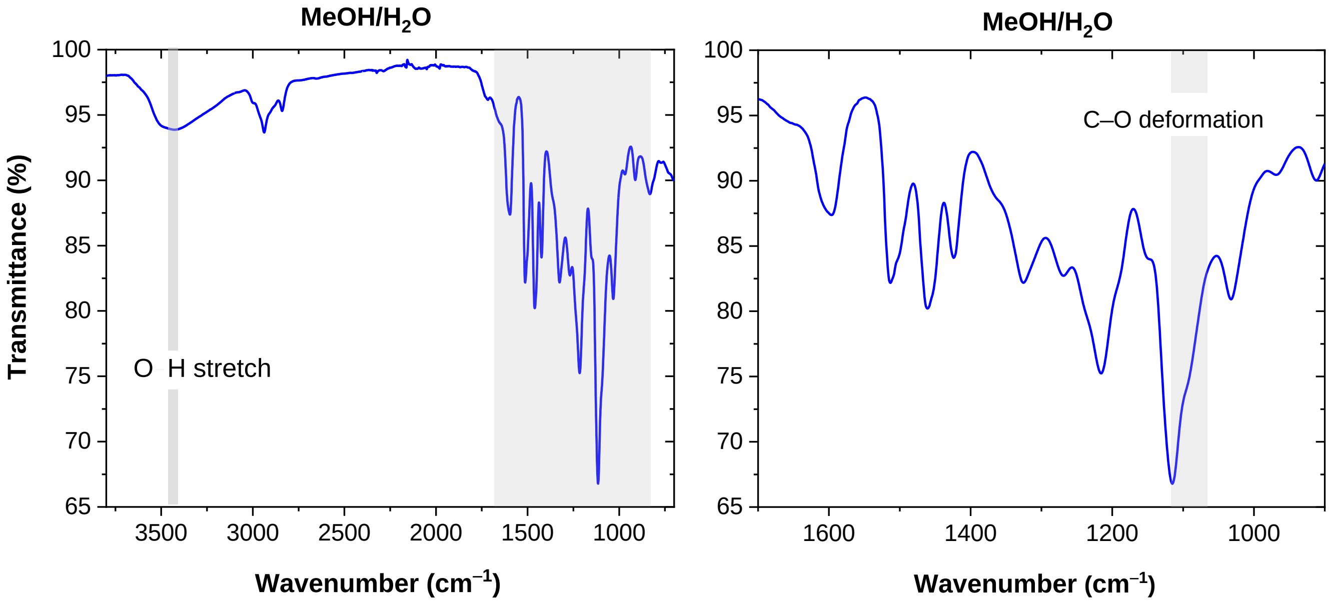

Figure S6. ATR-FTIR spectra of lignin isolated in MeOH/H_2O with the enlarged highlighted area of the original spectra.

Fractionation

Table S2. Lignin isolation in different solvents.[a]

Exp.	Solvent	Residue [%]	Lignin yield[b] [%]	Average *Mw* [Da]
1	MeOH	39.3	70.6	2150
2	EtOH	42.9	64.4	2050
3	MeOH/H_2O	43.7	76.0	2300
4	EtOH/H_2O	43.3	83.4	2700

[a] Reaction conditions: 180 ℃, 60 min, 1.0 % of H_2SO_4 (based on dry matter).
[b] Lignin content in beech wood was considered to be 24.4 %.[29]
[c] 75 vol% (m)ethanol/water.
